# Role of cationic drug-sensitive transport systems at the blood-cerebrospinal fluid barrier in *para*-tyramine elimination from rat brain

**DOI:** 10.1186/s12987-017-0087-9

**Published:** 2018-01-08

**Authors:** Shin-ichi Akanuma, Yuhei Yamazaki, Yoshiyuki Kubo, Ken-ichi Hosoya

**Affiliations:** Department of Pharmaceutics, Graduate School of Medicine and Pharmaceutical Sciences, University of Toyama, 2630 Sugitani, Toyama, 930-0194 Japan

**Keywords:** Biogenic amine, Blood-cerebrospinal fluid barrier, Blood–brain barrier, Choroid plexus, Clearance, Transporter, *para*-tyramine, *p*-TA

## Abstract

**Background:**

*para*-Tyramine (*p*-TA) is a biogenic amine which is involved in multiple neuronal signal transductions. Since the concentration of *p*-TA in dog cerebrospinal fluid (CSF) has been reported to be greater than that in plasma, it is proposed that clearance of cerebral *p*-TA is important for normal function. The purpose of this study was to examine the role of the blood–brain barrier and blood-cerebrospinal fluid barrier (BCSFB) in *p*-TA clearance from the brain.

**Methods:**

In vivo [^3^H]*p*-TA elimination from rat cerebral cortex and from CSF was examined after intracerebral and intracerebroventricular administration, respectively. To evaluate BCSFB-mediated *p*-TA transport, [^3^H]*p*-TA uptake by isolated rat choroid plexus and conditionally immortalized rat choroid plexus epithelial cells, TR-CSFB3 cells, was performed.

**Results:**

The half-life of [^3^H]*p*-TA elimination from rat CSF was found to be 2.9 min, which is 62-fold faster than that from rat cerebral cortex. In addition, this [^3^H]*p*-TA elimination from the CSF was significantly inhibited by co-injection of excess unlabeled *p*-TA. Thus, carrier-mediated *p*-TA transport process(es) are assumed to take part in *p*-TA elimination from the CSF. Since it is known that transporters at the BCSFB participate in compound elimination from the CSF, [^3^H]*p*-TA transport in ex vivo and in vitro models of rat BCSFB was examined. The [^3^H]*p*-TA uptake by isolated rat choroid plexus and TR-CSFB3 cells was time-dependent and was inhibited by unlabeled *p*-TA, indicating carrier-mediated *p*-TA transport at the BCSFB. The *p*-TA uptake by isolated choroid plexus and TR-CSFB3 cells was not reduced in the absence of extracellular Na^+^ and Cl^−^, and in the presence of substrates of typical organic cation transporters. However, this *p*-TA uptake was significantly inhibited by cationic drugs such as propranolol, imipramine, amantadine, verapamil, and pyrilamine. Moreover, *p*-TA uptake by TR-CSFB3 cells took place in an oppositely-directed H^+^ gradient manner. Therefore, this suggested that *p*-TA transport at the BCSFB involves cationic drug-sensitive transport systems which are distinct from typical plasma membrane organic cation transporters.

**Conclusion:**

Our study indicates that *p*-TA elimination from the CSF is greater than that from the cerebral cortex. Moreover, it is suggested that cationic drug-sensitive transport systems in the BCSFB participate in this *p*-TA elimination from the CSF.

## Background

*para*-Tyramine (*p*-TA) is one of the trace amines derived from tyrosine and present in fermented foods such as cheese [1, 2]. In the brain, *p*-TA acts as a neuromodulator, which supports neuronal actions by several neurotransmitters such as l-glutamate and norepinephrine [3, 4]. In addition, *p*-TA binds to trace amine-associated receptor 1, which is a G protein-coupled receptor and is a therapeutic target for schizophrenia [5]. It has been reported that, after activating the receptor, serotonergic and dopaminergic neuronal actions via serotonin receptor 1A and presynaptic dopamine D2 receptor, respectively, are reduced [6–10]. Since *p*-TA has a variety of roles in neuronal signal transduction, it is important to understand the homeostatic systems governing the *p*-TA concentration in the brain.

It is considered that the neural concentration of *p*-TA is maintained by a balance between production and clearance and there are reports that the activity of in vivo blood-to-brain transport of *p*-TA is low [11, 12]. In addition, Faraj et al. [13] have shown that the *p*-TA concentration in the cerebrospinal fluid (CSF) of dogs is approximately 2.6-fold lower than that in plasma although *p*-TA is produced in the brain. Therefore, it is conceivable that the *p*-TA clearance from the brain is relatively greater compared with that of *p*-TA production. Regarding the metabolic pathway of *p*-TA, it is known to be metabolized to octopamine and *p*-hydroxyphenylacetate via dopamine β-hydroxylase and monoamine oxidase, respectively [14]. Brain barriers are involved in the clearance of several neural compounds: the blood-brain barrier (BBB), which is formed by brain capillary endothelial cells, directly separates the brain from the circulating blood [15]. In addition, the cerebrospinal fluid (CSF) is separated from the circulating blood by the blood-CSF barrier (BCSFB) composed of choroid plexus epithelial cells [15]. Recent reports have shown that these barriers play a role in brain-to-blood transport of endogenous anionic and cationic compounds such as homovanillic acid and histamine [16, 17]. Therefore, there is a possibility that the BBB and/or BCSFB transport *p*-TA from the brain to the circulating blood and play a role in the *p*-TA clearance system in the brain.

These cells express a variety of plasma membrane transporters which contribute to compound transport across the BBB and BCSFB. In rat brain capillary endothelial cells, mRNAs of serotonin transporter [SERT/solute carrier (Slc) 6a4], organic cation transporter 1–3 (OCT1–3/Slc22a1–3), multidrug and toxin extrusion 1–2 (MATE1–2/Slc47a1–2), organic cation/carnitine transporter 1–2 (OCTN1–2/Slc22a4–5), and plasma membrane monoamine transporter (PMAT/Slc29a4) have been reported to be expressed [18, 19]. Among these transporters, it has been demonstrated that PMAT is involved in 1-methyl-4-phenylpyridinium (MPP^+^) efflux transport at the BBB, at least in part [18]. Regarding the BCSFB, the mRNA expression of these transporters has also been reported [18]. In addition, our previous studies have shown that PMAT and OCT3 are involved in the elimination of histamine and creatinine, respectively, from rat CSF [16, 20]. Taking these reports into consideration, it is possible that several cationic transporters at these brain barriers take part in the clearance of cationic compounds from the brain. It is known that *p*-TA is a substrate of plasma membrane transporters, such as SERT, OCT3, PMAT, and dopamine transporter (DAT/Slc6a3) [21–24] and, thus, it is hypothesized that *p*-TA in the brain is eliminated via these plasma membrane transporters at the brain barriers.

To increase our knowledge of the homeostatic mechanism(s) governing the cerebral *p*-TA concentration, the purpose of this study was to examine the role of the brain barriers in *p*-TA clearance from the brain. To examine *p*-TA elimination across the BBB, we used an intracerebral microinjection technique. To evaluate BCSFB-mediated *p*-TA efflux transport, in vivo intracerebroventricular administration and ex vivo transport studies using isolated choroid plexus were performed. In addition, the properties of *p*-TA uptake were examined using an in vitro model of rat BCSFB, conditionally immortalized rat choroid plexus epithelial cells (TR-CSFB3 cells).

## Methods

### Reagents

Butanol, *n* [1-^14^C] ([^14^C]*n*-butanol, 2 mCi/mmol), mannitol, d-[1-^14^C] ([^14^C]d-mannitol, 55 mCi/mmol), and tyramine hydrochloride, [ring-3,5-^3^H]-([^3^H]*p*-TA, 50 Ci/mmol) were obtained from American Radiolabeled Chemicals (St. Louis, MO, USA). All other reagents were commercially available.

### Animals

Wistar rats (male, 6-week-old, ~ 160 g) were purchased from Japan SLC (Hamamatsu, Japan) and maintained in a controlled environment.

### In vivo technique for evaluating *p*-TA elimination across the BBB [25]

Anesthetized rats, obtained by intraperitoneally injecting pentobarbital sodium solution (50 mg/kg), were fixed in a stereotaxic apparatus (SR-5R; Narishige, Tokyo, Japan). Physiological buffer A (0.5 µL; 122 mM NaCl, 25 mM NaHCO_3_, 10 mM d-glucose, and 10 mM 2-[4-(2-hydroxyethyl)-1-piperazinyl]ethansulfonic acid (HEPES)-NaOH, 3 mM KCl, 1.4 mM CaCl_2_, 1.2 mM MgSO_4_, 0.4 mM K_2_HPO_4_, pH 7.4) containing [^3^H]*p*-TA (120 nCi/0.5 μL, 2.0 μM) and [^14^C]d-mannitol (6.0 nCi/0.5 μL, 220 μM) was injected into the parietal cortex area two region of the brain over a period of 1 min. [^14^C]d-Mannitol, a BBB-impermeable marker, was used to normalize the actual injection volume. At a designated time, each rat was decapitated, and the ipsilateral cerebrum was removed. During the procedure, the behavior of rats and abnormal morphology of the brain was monitored, and there was no apparent change in the anesthetized conditions and brain structure. The tissue was dissolved in 2 N NaOH (2 mL) at 55 °C for 3 h and then mixed with 10 mL Hionic-Fluor (PerkinElmer, Boston, MA, USA). The radioactivity was measured in a liquid scintillation counter equipped with an appropriate crossover correction for ^3^H and ^14^C (LSC-6101; Hitachi-Aloka Medical, Tokyo, Japan). The percentage of [^3^H]*p*-TA remaining in the ipsilateral cerebrum (%) was obtained from Eq. 1.

1 $$\left[ {^{ 3} {\text{H}}} \right]p{\text{-TA}}\;{\text{remaining}}\;{\text{in}}\;{\text{the}}\;{\text{ipsilateral}}\;{\text{cerebrum}}\;\left( \% \right) = \frac{\left[ ^{ 3} {\text{H}} \right]p{\text{-TA}} /\left[ ^{ 1 4} {\text{C}} \right]{{D}}{\text{-mannitol}}\;{\text{in}}\;{\text{the}}\;{\text{cerebrum}}}{\left[ ^{ 3} {\text{H}} \right]p{\text{-TA}}/\left[ ^{ 1 4} {\text{C}} \right]{{D}}{\text{-mannitol}}\;{\text{in}}\;{\text{the}}\;{\text{injectate}}} \times 100$$

### Lateral ventricular micro-administration [26]

Rats were anesthetized by intraperitoneally injecting pentobarbital solution (50 mg/kg), and the head was fixed in the SR-5R (Narishige). A hole was drilled in the skull, 1.5 mm to the left and 0.5 mm posterior to the bregma, into which a needle was fixed as a cannula for injection. Then, 10 μL buffer A containing [^3^H]*p*-TA (400 nCi) and [^14^C]d-mannitol (5 nCi), with or without unlabeled 75 mM *p*-TA, was injected into the left lateral ventricle over a period of 20 s. At designated times, CSF (50 μL) was withdrawn by cisternal puncture and the levels of ^3^H and ^14^C in the CSF and injectate were measured in a liquid scintillation counter (LSC-6101; Hitachi-Aloka Medical). The rats exhibited no abnormal behavior during this technique.

The kinetic parameters of this study were obtained using a one-compartmental model (Eq. 2), where *C*_CSF_(*t*), *k*_e,CSF_, and *V*_d,CSF_ are the concentration in CSF at time *t*, the elimination rate constant from the CSF, and the distribution volume in the CSF, respectively, of either [^3^H]*p*-TA or [^14^C]d-mannitol.

2 $$C_{\text{CSF}} \left( t \right) = \frac{\text{Dose}}{{V_{{{\text{d}},{\text{CSF}}}} }} \times { \exp }\left( { - k_{{{\text{e}},{\text{CSF}}}} \times t} \right)$$

In addition, “*C*_CSF_(t)/dose × 100” is the percentage of the residual concentration of the compound in the CSF normalized by the amount of injectate (% of dose/mL CSF). By multiplying *k*_e_ by *V*_d,CSF_, the apparent elimination clearance from the CSF (*CL*_CSF_) was obtained.

### Transport study using choroid plexus [27]

Rats were decapitated and the choroid plexus in the lateral ventricles was isolated. After preincubation of the choroid plexus in buffer A for 1 min at 37 °C, a transport reaction was initiated by applying buffer A containing 1.0 μCi [^3^H]*p*-TA and 0.05 μCi [^14^C]*n*-butanol, a marker of water space in the choroid plexus [28], in the absence or presence of unlabeled compounds. Na^+^-free or Cl^−^-free buffer was prepared by equimolar replacement of NaCl and NaHCO_3_ with lithium chloride and potassium bicarbonate or that of NaCl, KCl, and CaCl_2_ with sodium gluconate, potassium gluconate, and calcium gluconate, respectively. At designated times, the uptake buffer was removed, and the choroid plexus was solubilized by adding 3 N KOH. A liquid scintillation cocktail (Hionic-Fluor; PerkinElmer) was added to the sample, and ^3^H- and ^14^C-radioactivities were measured using an LSC-6101 (Hitachi-Aloka Medical).

### Uptake by conditionally immortalized rat choroid plexus epithelial cells [29]

TR-CSFB3 cells were cultured at 33 °C as described previously. Kitazawa et al. have determined the apical localization of Na^+^, K^+^-ATPase from an immunocytochemical study using TR-CSFB3 cells cultured onto a glass slide [29]. It has also been demonstrated that the apical-to-cell uptake capacity of [^3^H]l-proline in TR-CSFB3 cells seeded onto a Transwell^®^ was similar to the uptake value of [^3^H]l-proline in TR-CSFB3 cells seeded onto a culture plate [29]. The cells were plated onto collagen I-coated 24-well culture plates (BD-Biosciences, Franklin Lakes, NJ, USA) at a density of 1.0 × 10^5^ cells/well and cultured for 2 days at 33 °C. At 37 °C, the cells were washed three times with buffer A and 200 μL [^3^H]*p*-TA-containing buffer A (0.75 μCi/mL) was applied. Na^+^- or Cl^−^-free buffer A was prepared as described in the previous section. To stop the uptake reaction, cells were rinsed three times with 4 °C buffer A. The cells were subsequently solubilized with 1 N NaOH solution and incubated at room temperature for 12 h. After neutralization with 1 N HCl, the cellular protein and [^3^H]*p*-TA-derived radioactivity were determined using a detergent-compatible protein assay kit (BIO-RAD, Hercules, CA, USA) and liquid scintillation counting, respectively.

### Data analyses

The distribution volume of [^3^H]*p*-TA in isolated rat choroid plexus and TR-CSFB3 cells, namely the tissue/medium ratio and cell/medium ratio, respectively, was obtained from the following equations (Eqs. 3, 4).

3 $${{\text{Tissue}} \mathord{\left/ {\vphantom {{\text{Tissue}} {\text{medium ratio}}}} \right. \kern-0pt} {\text{medium ratio}}}\;\left( {{{\upmu{\text{L}}} \mathord{\left/ {\vphantom {{\upmu{\text{L}}} {\upmu{\text{L}}\;{\text{ChP}}}}} \right. \kern-0pt} {\upmu{\text{L}}\;{\text{ChP}}}}} \right) = \frac{{{{\left[ {^{ 3} {\text{H}}} \right]p{\text{-TA}}\;{\text{in}}\;{\text{the}}\;{\text{choroid}}\;{\text{plexus}}\;\left( {{\text{dpm}}/{\text{ChP}}} \right)} \mathord{\left/ {\vphantom {{\left[ {^{ 3} {\text{H}}} \right]p{\text{-TA}}\;{\text{in}}\;{\text{the}}\;{\text{choroid}}\;{\text{plexus}}\;\left( {{\text{dpm}}/{\text{ChP}}} \right)} {\left[ {^{ 3} {\text{H}}} \right]p{\text{-TA}}\;{\text{concentration}}\;{\text{in}}\;{\text{buffer}}\;({\text{dpm}}/\upmu{\text{L}})}}} \right. \kern-0pt} {\left[ {^{ 3} {\text{H}}} \right]p{\text{-TA}}\;{\text{concentration}}\;{\text{in}}\;{\text{buffer}}\;({\text{dpm}}/\upmu{\text{L}})}}}}{{{{\left[ {^{ 1 4} {\text{C}}} \right]n{\text{-Butanol}}\;{\text{in}}\;{\text{the}}\;{\text{choroid}}\;{\text{plexus}}\;\left( {{\text{dpm}}/{\text{ChP}}} \right)} \mathord{\left/ {\vphantom {{\left[ {^{ 1 4} {\text{C}}} \right]n{\text{-Butanol}}\;{\text{in}}\;{\text{the}}\;{\text{choroid}}\;{\text{plexus}}\;\left( {{\text{dpm}}/{\text{ChP}}} \right)} {\left[ {^{ 1 4} {\text{C}}} \right]n{\text{-Butanol}}\;{\text{concentration}}\;{\text{in}}\;{\text{buffer}}\;({\text{dpm}}/\mu {\text{L}})}}} \right. \kern-0pt} {\left[ {^{ 1 4} {\text{C}}} \right]n{\text{-Butanol}}\;{\text{concentration}}\;{\text{in}}\;{\text{buffer}}\;({\text{dpm}}/\mu {\text{L}})}}}}$$

4 $${{\text{Cell}} \mathord{\left/ {\vphantom {{\text{Cell}} {\text{medium ratio}}}} \right. \kern-0pt} {\text{medium ratio}}}\;\left( {{{\upmu{\text{L}}} \mathord{\left/ {\vphantom {{\upmu{\text{L}}} {{\text{mg}}\;{\text{protein}}}}} \right. \kern-0pt} {{\text{mg}}\;{\text{protein}}}}} \right) = \frac{{{\text{Intracellular }}\left[ {^{ 3} {\text{H}}} \right]p{\text{-TA}}\;{\text{per}}\;{\text{cellular}}\;{\text{protein}}\;{\text{amount}}\;\left( {{\text{dpm}}/{\text{mg}}\;{\text{protein}}} \right)}}{{\left[ {^{ 3} {\text{H}}} \right]p{\text{-TA}}\;{\text{concentration}}\;{\text{in}}\;{\text{buffer}}\;({\text{dpm}}/\upmu{\text{L}})}}$$

The initial uptake clearance of [^3^H]*p*-TA by isolated rat choroid plexus and TR-CSFB3 cells was calculated using Eqs. 5 and 6, respectively.

5 $${\text{Tissue}}/{\text{medium ratio}}\;\left( {{{\upmu{\text{L}}} \mathord{\left/ {\vphantom {{\upmu{\text{L}}} {\upmu{\text{L}}\;{\text{ChP}}}}} \right. \kern-0pt} {\upmu{\text{L}}\;{\text{ChP}}}}} \right) = {\text{Initial uptake clearance }}\left( {{{\upmu{\text{L}}} \mathord{\left/ {\vphantom {{\upmu{\text{L}}} {\left( {{ \hbox{min} }\;\upmu{\text{L ChP}}} \right)}}} \right. \kern-0pt} {\left( {{ \hbox{min} }\;\upmu{\text{L ChP}}} \right)}}} \right) \times {\text{Time}}\;\left( { \hbox{min} } \right) + {\text{Initial}}\;{\text{distribution volume}}\;\left( {{{\upmu{\text{L}}} \mathord{\left/ {\vphantom {{\upmu{\text{L}}} {\upmu{\text{L}}\;{\text{ChP}}}}} \right. \kern-0pt} {\upmu{\text{L}}\;{\text{ChP}}}}} \right)$$

6 $${{\text{Cell}} \mathord{\left/ {\vphantom {{\text{Cell}} {\text{medium ratio}}}} \right. \kern-0pt} {\text{medium ratio}}}\left( {{{\upmu{\text{L}}} \mathord{\left/ {\vphantom {{\upmu{\text{L}}} {{\text{mg}}\;{\text{protein}}}}} \right. \kern-0pt} {{\text{mg}}\;{\text{protein}}}}} \right) = {\text{Initial}}\;{\text{uptake}}\;{\text{clearance}}\;\left( {{{\upmu{\text{L}}} \mathord{\left/ {\vphantom {{\upmu{\text{L}}} {\left( {{ \hbox{min} }\;{\text{mg protein}}} \right)}}} \right. \kern-0pt} {\left( {{ \hbox{min} }\;{\text{mg protein}}} \right)}}} \right) \times {\text{Time}}\;\left( { \hbox{min} } \right) + {\text{Initial}}\;{\text{distribution}}\;{\text{volume}}\;\left( {{{\upmu{\text{L}}} \mathord{\left/ {\vphantom {{\upmu{\text{L}}} {{\text{mg}}\;{\text{protein}}}}} \right. \kern-0pt} {{\text{mg}}\;{\text{protein}}}}} \right)$$

The equation was fitted using the nonlinear least-squares regression analysis program, MULTI [30]. To examine the initial uptake process, a linear correlation analysis was performed. The coefficient of determination (*r* ^2^) of [^3^H]*p*-TA uptake by isolated rat choroid plexus from 0.25 min to 1.0 min was found to be 0.981, which was greater than that from 0.25 min to 2.0 min (*r* ^2^ = 0.822). This suggested that the initial [^3^H]*p*-TA uptake by isolated rat choroid plexus is reflected in the data up to 1 min. Regarding the uptake of [^3^H]*p*-TA by TR-CSFB3 cells, the *r* ^2^ of the [^3^H]*p*-TA uptake from 0.25 min to 2.0 min was calculated as 0.989, which is similar to that of [^3^H]*p*-TA uptake by isolated rat choroid plexus from 0.25 min to 1.0 min and is close to 1. This result suggests that the cell/medium ratio at all time points reflects the initial [^3^H]*p*-TA uptake process.

The kinetic parameters for *p*-TA uptake by TR-CSFB3 cells were obtained from Eq. 7:

7 $$V = {{\left( {V_{ \hbox{max} } \times \left[ {\text{S}} \right]} \right)} \mathord{\left/ {\vphantom {{\left( {V_{ \hbox{max} } \times \left[ {\text{S}} \right]} \right)} {\left( {K_{\text{m}} + \, \left[ {\text{S}} \right]} \right)}}} \right. \kern-0pt} {\left( {K_{\text{m}} + \, \left[ {\text{S}} \right]} \right)}} + K_{\text{d}} \times \left[ {\text{S}} \right]$$

where *V* is the uptake rate of *p*-TA, [S] is the *p*-TA concentration in buffer, *K*_m_ is the Michaelis–Menten constant, and *K*_d_ is the non-saturable transport clearance. The equation was fitted using the MULTI program [30]. The kinetic parameters are presented as the mean ± standard deviation (S.D.). Other data represent the mean ± standard error of the mean (S.E.M.). The statistical significance of differences between the means was determined using the unpaired Student’s *t* test for two groups and one-way analysis of variance followed by Dunnett’s test for more than three groups.

## Results

### Evaluation of *p*-TA elimination from rat cerebral cortex

**Fig. 1:**
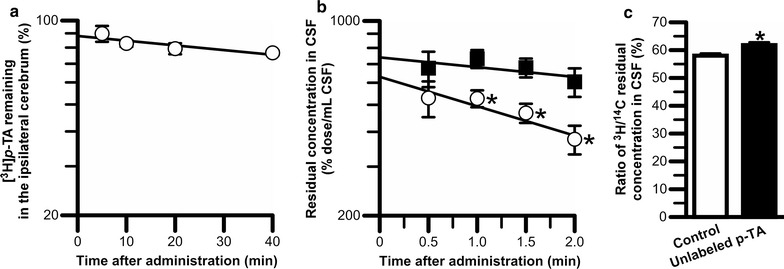
In vivo analyses of *p*-TA elimination from rat brain or cerebrospinal fluid. **a** Time profile of [^3^H]*p*-TA remaining in the ipsilateral cerebrum after injection into rat cerebral cortex. A mixture of [^3^H]*p*-TA (120 nCi) and [^14^C]d-mannitol (6 nCi) dissolved in 0.5 μL buffer A was injected. The solid line was obtained by MULTI analysis. Each point represents the mean ± S.E.M. (*n* = 3). **b** Time profile of the residual CSF concentration (% dose × 100) of [^3^H]*p*-TA (open circles) and [^14^C]d-mannitol (closed squares) after administration into rat lateral ventricles and sampled from the cisterna magna. Buffer A containing [^3^H]*p*-TA (400 nCi/10 μL) and [^14^C]d-mannitol (5 nCi/10 μL) was injected into rat lateral ventricles. The solid line was obtained by MULTI analysis. Each point represents the mean ± S.E.M. (*n* = 3–4). *p < 0.05, significantly different from the value for [^14^C]d-mannitol. **c** Increase in the residual CSF concentration of [^3^H]*p*-TA at 2 min normalized to that of [^14^C]d-mannitol by simultaneous administration of unlabeled 75 mM *p*-TA. Each column represents the mean ± S.E.M. (*n* = 3–5). *p < 0.05, significantly different from the control

The time-course of the percentage [^3^H]*p*-TA remaining in rat ipsilateral cerebrum at 5, 10, 20, and 40 min after micro-administration into the parietal cortex area 2 is shown in Fig. 1a. There was no significant difference between the values at 5, 10, 20, and 40 min (*p* = 0.143), although a tendency for a time-dependent decrease was observed. As a reference value, the half-life (*t*_1/2_) of the percentage of cerebral [^3^H]*p*-TA remaining after microinjection into the cerebral cortex was calculated to be 178 ± 68 min.

### In vivo *p*-TA elimination from rat CSF

Figure 1b shows the time profile of “*C*_CSF_(t)/dose × 100” values of [^3^H]*p*-TA and [^14^C]d-mannitol after intracerebroventricular injection. The residual concentration in CSF of [^3^H]*p*-TA at 1.0, 1.5, and 2.0 min was significantly lower than that of [^14^C]d-mannitol. [^3^H]*p*-TA was eliminated from the CSF with a *t*_1/2_ of 2.85 ± 0.83 min, which is 3.0-fold faster than the *t*_1/2_ of [^14^C]d-mannitol. From the *V*_d,CSF_ and *k*_e,CSF_ values of [^3^H]*p*-TA (*V*_d,CSF_, 159 ± 16 μL/rat; *k*_e,CSF_, 2.43 × 10^−1^ ± 0.72 × 10^−1^ min^−1^), the *CL*_CSF_ of [^3^H]*p*-TA was found to be 38.6 ± 12.0 μL/rat. The kinetic parameters for [^14^C]d-mannitol, *V*_d,CSF_, *k*_e,CSF_, and *CL*_CSF_, were calculated to be 135 ± 11 μL/rat, 8.07 × 10^−2^ ± 6.09 × 10^−2^ min^−1^, and 10.9 ± 8.3 μL/rat, respectively. To examine the possible contribution of carrier-mediated *p*-TA elimination system(s) from the CSF, the effect of unlabeled excess *p*-TA on the concentration ratio of [^3^H]*p*-TA and [^14^C]d-mannitol was examined. The [^3^H]*p*-TA/[^14^C]d-mannitol residual concentration ratio at 2 min was significantly (1.1-fold) increased by co-administration of 75 mM unlabeled *p*-TA into the rat cerebroventricle compared with that in the control (Fig. 1c), indicating that [^3^H]*p*-TA elimination from rat CSF was inhibited by excess unlabeled *p*-TA.

### [^3^H]*p*-TA uptake by isolated rat choroid plexus

**Fig. 2:**
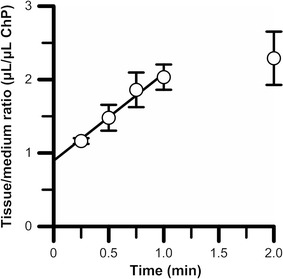
Time-dependent uptake of [^3^H]*p*-TA by isolated rat choroid plexus. The choroid plexus was incubated with [^3^H]*p*-TA (1 μCi/sample, 1 μM) and [^14^C]*n*-butanol (0.05 μCi/sample, 250 nM) at 37 °C. The solid line was obtained by MULTI analysis. Each point represents the mean ± S.E.M. (*n* = 3–6)

In order to examine whether *p*-TA was eliminated from the CSF across the BCSFB, a [^3^H]*p*-TA transport study using isolated rat choroid plexus was performed. [^3^H]*p*-TA was time-dependently taken up into the isolated rat choroid plexus with an initial uptake rate and an initial distribution volume of 1.19 ± 0.31 μL/(min μL ChP) and 0.895 ± 0.223 μL/μL ChP, respectively (Fig. 2). As shown in Table 1, 10 mM unlabeled *p*-TA significantly inhibited this [^3^H]*p*-TA uptake by 34%. The absence of extracellular Na^+^ or Cl^−^ did not significantly affect the [^3^H]*p*-TA uptake by isolated rat choroid plexus (Table 1), indicating that Na^+^ and/or Cl^−^-cotransport systems are not involved in *p*-TA uptake by the rat choroid plexus. To examine *p*-TA transport mechanism(s) on the choroid plexus, the compound/drug-sensitivities to [^3^H]*p*-TA uptake were examined (Table 1). Several cationic drugs, such as propranolol, pyrilamine, amantadine, imipramine, and verapamil, significantly inhibited [^3^H]*p*-TA uptake by the isolated rat choroid plexus by more than 29%. Among typical SLC transporter substrates/inhibitors, choline exhibited 28% inhibition of [^3^H]*p*-TA uptake. However, [^3^H]*p*-TA uptake by the isolated rat choroid plexus was not significantly altered in the presence of serotonin (a substrate of SERT), MPP^+^ (a typical substrate of OCT and PMAT), tetraethylammonium (TEA; a typical substrate of OCT, OCTN, and MATE), and *p*-aminohippurate (PAH; a typical substrate of organic anion transporter, OAT).

**Table 1:** Effect of Na^+^- or Cl^−^-replacement, and co-presence of compounds on [^3^H]*p*-TA uptake by isolated rat choroid plexus

Compounds	Concentration (mM)	Percentage of control
*Na* ^+^ */Cl* ^−^-*replacement*		
Control		100 ± 5
Na^+^-free		93.6 ± 3.9
Cl^−^-free		98.4 ± 5.8
*Co-presence of compounds*		
Control		100 ± 9
*p*-TA	10	65.9 ± 4.6**
Propranolol	10	61.0 ± 5.4**
Pyrilamine	10	61.2 ± 0.8**
Amantadine	10	61.4 ± 0.6**
Imipramine	1	67.4 ± 1.1*
Verapamil	1	70.8 ± 1.1*
Choline	10	72.3 ± 4.6*
Serotonin	10	79.6 ± 2.2
MPP^+^	10	84.7 ± 6.3
TEA	10	93.0 ± 3.1
PAH	10	95.4 ± 5.1

[^3^H]*p*-TA uptake by isolated rat choroid plexus (1 µCi/sample) was performed at 37 °C for 45 s in the absence (control) or presence of Na^+^ and Cl^−^ without (control) or with unlabeled compounds. Each value represents the mean ± S.E.M. (*n* = 3–8)

*MPP* ^*+*^ 1-methyl-4-phenylpyridinium, *PAH p*-aminohippurate, *TEA* tetraethylammonium, *p-TA para*-tyramine

*p < 0.05 and **p < 0.01, significantly different from control

### Characteristics of [^3^H]*p*-TA uptake by TR-CSFB3 cells

**Fig. 3:**
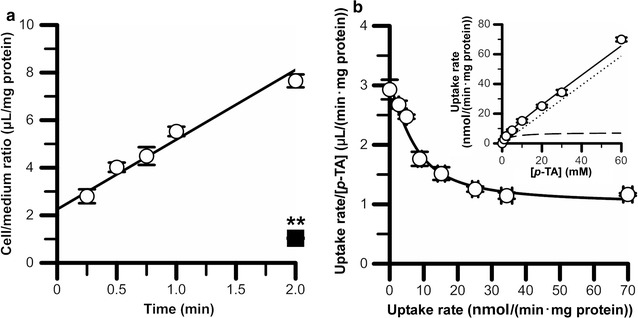
Time-, temperature-, and concentration-dependence of [^3^H]*p*-TA uptake by TR-CSFB3 cells. **a** [^3^H]*p*-TA uptake (0.15 μCi/well, 19 nM) was measured at 37 °C (open circles) and 4 °C (closed square) for indicated times. The solid line was obtained by MULTI analysis. Each point represents the mean ± S.E.M. (*n* = 3). **p < 0.01, significantly different from [^3^H]*p*-TA uptake at 37 °C for 2 min. **b** *p*-TA uptake, over the concentration range 20 nM–60 mM, was measured at 37 °C for 2 min. The data were subjected to Eadie–Scatchard analysis in addition to Michaelis–Menten kinetics (inset). The solid, dashed, and dotted lines, which were fitted by the MULTI program, represent overall, saturable, and non-saturable transport, respectively. Each point represents the mean ± S.E.M. (*n* = 3)

To clarify the characteristics of *p*-TA transport at the BCSFB, a [^3^H]*p*-TA transport study using an in vitro model of rat BCSFB, TR-CSFB3 cells, was performed. TR-CSFB3 cells exhibited time-dependent [^3^H]*p*-TA uptake with an initial uptake rate and an initial distribution volume of 2.93 ± 0.32 μL/(min mg protein) and 2.25 ± 0.24 μL/mg protein (Fig. 3a, open circles). At 2 min, [^3^H]*p*-TA uptake by TR-CSFB3 cells at 4 °C was significantly reduced by 86% (Fig. 3a, closed square). In addition, concentration-dependent *p*-TA uptake by TR-CSFB3 cells consisted of saturable and non-saturable components with apparent *K*_m_, *V*_max_ and *K*_d_ values of 3.48 ± 0.83 mM, 7.26 ± 1.61 nmol/(min mg protein), and 9.78 × 10^−1^ ± 0.66 × 10^−1^ μL/(min mg protein), respectively (Fig. 3b). Moreover, 10 mM *p*-TA significantly inhibited the [^3^H]*p*-TA uptake by 48% (Table 2). [^3^H]*p*-TA uptake by TR-CSFB3 cells was significantly inhibited by more than 30% in the presence of several kinds of cationic drugs/compounds, such as propranolol, imipramine, amantadine, nicotine, desipramine, pyrilamine, and verapamil. In contrast, this [^3^H]*p*-TA uptake was not significantly altered by l-carnitine (a typical substrate of OCTN), pyrimethamine (a substrate of MATE), norepinephrine (a substrate of norepinephrine transporter), PAH, serotonin, and cimetidine (a substrate of OCT and MATE). In addition, the [^3^H]*p*-TA uptake by TR-CSFB3 cells was significantly increased, but not decreased, by the co-presence of tyrosine (a precursor of *p*-TA), choline (a substrate of OCT), MPP^+^, and TEA (Table 2).

**Table 2:** Effect of Na^+^- or Cl^−^-replacement, and co-presence of compounds on [^3^H]*p*-TA uptake by TR-CSFB3 cells

Compounds	Concentration (mM)	Percentage of control
*Na* ^+^ */Cl* ^−^-*replacement*		
Control		100 ± 3
Na^+^-free		153 ± 8**
Cl^−^-free		103 ± 4
*Co-presence of compounds*		
Control		100 ± 1
*p*-TA	10	51.8 ± 3.7**
Propranolol	10	29.0 ± 4.2**
Imipramine	10	31.5 ± 2.9**
Amantadine	10	45.3 ± 2.4**
Nicotine	10	52.2 ± 3.8**
Desipramine	10	63.4 ± 1.9**
Pyrilamine	10	65.4 ± 10.8**
Verapamil	3.0	69.6 ± 6.7*
l-Carnitine	10	75.0 ± 5.7
Norepinephrine	10	96.1 ± 7.3
PAH	10	110 ± 6
Serotonin	10	114 ± 5
Cimetidine	10	115 ± 2
Tyrosine	10	140 ± 17**
Choline	10	150 ± 6**
MPP^+^	10	155 ± 19**
TEA	10	168 ± 13**
Control (1% DMSO)		100 ± 5
Pyrimethamine (1% DMSO)	0.2	91.0 ± 7.3

[^3^H]*p*-TA uptake by TR-CSFB3 cells (0.15 µCi/well) was performed at 37 °C for 2 min in the absence (control) or presence of Na^+^ and Cl^−^ without (control) or with unlabeled compounds. Each value represents the mean ± S.E.M. (*n* = 3)

*DMSO* dimethyl sulfoxide, *MPP* ^*+*^ 1-methyl-4-phenylpyridinium, *PAH p*-aminohippurate, *TEA* tetraethylammonium, *p-TA para*-tyramine

*p < 0.05 and **p < 0.01, significantly different from control

**Fig. 4:**
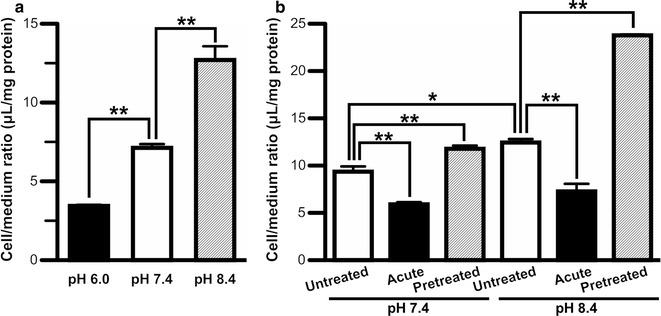
Extra- and intra-cellular pH dependence of [^3^H]*p*-TA uptake by TR-CSFB3 cells. **a** [^3^H]*p*-TA uptake (0.15 μCi/well) at pH 6.0, pH 7.4, and pH 8.4 was tested at 37 °C for 2 min. Each column represents the mean ± S.E.M. (*n* = 3). **p < 0.01, significant difference between the groups. **b** TR-CSFB3 cells were preincubated with buffer A at pH 7.4 or pH 8.4 in the absence (untreated and acute) or presence (pretreated) of 30 mM ammonium chloride for 20 min. After removal of the buffer, the cells were incubated with [^3^H]*p*-TA (0.15 μCi/well) at pH 7.4 or pH 8.4 in the absence (untreated and pretreated) or presence (acute) of 30 mM ammonium chloride at 37 °C for 2 min. Each column represents the mean ± SEM (*n* = 3–6). *p < 0.05 and **p < 0.01, significant difference between the groups

The extracellular inorganic anion sensitivity of [^3^H]*p*-TA uptake by TR-CSFB3 cells was examined and was found not to be altered in the absence of extracellular Cl^−^, whereas it was significantly increased, but not decreased, in the absence of extracellular Na^+^ (Table 2). This result indicates that Na^+^- and Cl^−^-cotransporters are not involved in *p*-TA transport into the choroid plexus. The [^3^H]*p*-TA uptake by TR-CSFB3 cells was significantly reduced by 50% at an extracellular pH of 6.0 (Fig. 4a, closed column), and increased 1.8-fold at an extracellular pH of 8.4 (Fig. 4a, hatched column). In order to examine the intracellular pH effect, TR-CSFB3 cells were treated with ammonium chloride, since the intracellular pH is increased by treatment with ammonium chloride (acute), and subsequent removal of ammonium chloride (pretreated) reduces the intracellular pH [31]. An increase in the intracellular pH significantly reduced [^3^H]*p*-TA uptake by TR-CSFB3 cells at pH 7.4 and pH 8.4 by 37% and 42%, respectively (Fig. 4b, acute and closed column), compared with respective untreated conditions (Fig. 4b, untreated and open column). In contrast, a reduction in intracellular pH increased [^3^H]*p*-TA uptake by TR-CSFB3 cells to 126% and 191% at pH 7.4 and pH 8.4, respectively (Fig. 4b, pretreated and hatched column), relative to the respective untreated conditions.

## Discussion

In this study, *p*-TA elimination from the brain and CSF was investigated in rats (Fig. 1). To clarify the involvement of the BCSFB in *p*-TA elimination from the CSF, a *p*-TA transport study using isolated rat choroid plexus, which forms the BCSFB, was carried out (Fig. 2 and Table 1). Since carrier-mediated transport properties were observed in this study using rat choroid plexus, *p*-TA transport characteristics were examined in in vitro rat BCSFB model cells (Figs. 3, 4, and Table 2).

The percentage of [^3^H]*p*-TA remaining in the ipsilateral cerebrum tended to be reduced in a time-dependent manner (Fig. 1a), although there was no significant difference between the values at examined time points. It is known that *p*-TA is converted via enzymes in neural cells to several metabolites, such as octopamine [14]. Thus, it is suggested that *p*-TA and its metabolites are eliminated to a degree from the brain across the BBB. However, this *t*_1/2_, as a reference record (178 min), was 62-fold longer than that of the residual concentration of [^3^H]*p*-TA in the CSF (Fig. 1b). These results show that *p*-TA elimination from the CSF makes a major contribution to cerebral *p*-TA clearance relative to that from the brain parenchyma or interstitial fluid. In addition, the *CL*_CSF_ for [^3^H]*p*-TA was 3.5-fold higher than that of [^14^C]d-mannitol, a marker compound for bulk flow of CSF [26, 32]. Taking the inhibitory effect of *p*-TA elimination from the CSF by excess unlabeled *p*-TA (Fig. 1c) into consideration, this suggests that carrier-mediated transport system(s), which are distinct from CSF bulk flow, are involved in *p*-TA elimination from the CSF.

After intracerebroventricular administration, it is possible that [^3^H]*p*-TA is distributed to the brain parenchyma and/or taken up into ependymal cells and choroid plexus epithelial cells [33]. It is known that transporters at the BCSFB participate in the elimination of several compounds from the CSF [15]. We have shown that [^3^H]*p*-TA was time-dependently taken up into ex vivo isolated rat choroid plexus (Fig. 2) and in vitro choroid plexus epithelial cells, which form the BCSFB (Fig. 3). In addition, this [^3^H]*p*-TA uptake was significantly inhibited by unlabeled 10 mM *p*-TA (Tables 1, 2). Since the initial distribution volume of *p*-TA was found to be 159 μL (Fig. 1b), the concentration in rat CSF of unlabeled *p*-TA after a 10 μL intracerebroventricular microinjection of 75 mM unlabeled *p*-TA was calculated to be 4.7 mM, which is similar to that in the in vitro self-inhibition studies. Therefore, it is suggested that some transport systems at the BCSFB take part in carrier-mediated elimination of *p*-TA from the CSF.

Since the apical membrane of the choroid plexus faces the [^3^H]*p*-TA-containing buffer in the uptake study using isolated choroid plexus, it appears that the characteristics of [^3^H]*p*-TA uptake by rat choroid plexus reflects the CSF-to-cell transport direction at the BCSFB. In this study, the choroid plexus from the lateral ventricle was used in the *p*-TA transport study. Although there are known regional differences in physiological and transport functions between the choroid plexuses from the lateral, third, and fourth ventricles [34, 35], Ogawa et al. [26] have reported that the in vivo uptake rate which is extrapolated from in vitro benzylpenicillin transport in rat choroid plexus from the lateral ventricle, is in good agreement with that of saturable in vivo elimination of benzylpenicillin from the CSF. *p*-TA transport across the apical membrane of the isolated rat choroid plexus was found to be 7.14 μL/(min rat) since the total volume of rat choroid plexus has been reported to be 6 μL (7.14 μL/(min rat) = 1.19 μL/μL ChP × 6 μL/rat) [26]. Regarding the polarity of TR-CSFB3 cells after seeding onto a culture plate and glass slide, it has been reported that membrane protein localization onto the plasma membrane of choroid plexus epithelial cells is mostly retained [29]. As the surface area of TR-CSFB3 cells and rat lateral ventricle choroid plexus epithelium has been reported to be 20 cm^2^/mg protein [18] and 75 cm^2^/rat [36], respectively, the initial *p*-TA uptake clearance estimated from the uptake study using TR-CSFB3 cells was calculated to be 11.0 μL/(min rat) (= 2.93 μL/(min mg protein) ÷ 20 cm^2^/mg protein × 75 cm^2^/rat). This estimated initial *p*-TA uptake clearance from the transport study using TR-CSFB3 cells is consistent with that using isolated rat choroid plexus. Taking these findings into consideration, there is a high possibility that the characteristics of *p*-TA uptake by TR-CSFB3 cells reflect the *p*-TA transport properties of the apical membrane of the BCSFB.

These BCSFB-mediated [^3^H]*p*-TA uptake clearance values obtained from the uptake studies of isolated rat choroid plexus (7.14 μL/(min rat) and TR-CSFB3 cells (11.0 μL/(min rat)) were 18.5 and 28.5% of the in vivo total [^3^H]*p*-TA elimination clearance from rat CSF (38.6 μL/(min rat), Fig. 1b). As the elimination clearance of [^14^C]d-mannitol from rat CSF was found to be 10.9 μL (Fig. 1b), 28.2% of the total [^3^H]*p*-TA clearance would reflect the CSF bulk flow and diffusion to the brain parenchyma. As the other pathways for *p*-TA elimination from the CSF, the remainder of which corresponds to (43.3–53.5%), the incorporation of *p*-TA into the neural cells facing the cerebroventricles, including the ependymal cells, is a possibility. Further analysis to check the radioactivities in choroid plexus and neural cells surrounding the cerebroventricle could help these cells contribute to *p*-TA elimination from the CSF. Nevertheless, it is considered that the BCSFB is involved in the carrier-mediated *p*-TA elimination from the CSF, at least in part, since an inhibitory effect of unlabeled excess *p*-TA on [^3^H]*p*-TA uptake by isolated rat choroid plexus (Table 1) and TR-CSFB3 cells (Table 2) was observed.

*p*-TA uptake by TR-CSFB3 cells exhibited saturable and non-saturable kinetics (Fig. 3b). The clearance of the saturable process of *p*-TA transport (*V*_max_/*K*_m_) was found to be 2.09 μL/(min mg protein), which is 2.1-fold higher than that of the non-saturable process [*K*_d_, 0.978 μL/(min mg protein)]. This result indicates that the transporter-mediated process makes a major contribution to apical *p*-TA transport at the BCSFB. It has been reported that human OCT3 and PMAT accept *p*-TA as a substrate with a *K*_m_ of 0.281 and 283 μM, respectively [21, 22]. However, the *K*_m_ value of *p*-TA uptake by TR-CSFB3 cells (3.48 mM) was inconsistent with these values. mRNA expression of OCT1–2, OCTN1–2, and MATE in addition to OCT3 and PMAT as typical organic cation transporters has been reported [18] but no attenuation of [^3^H]*p*-TA transport into TR-CSFB3 cells and/or isolated rat choroid plexus was observed in the presence of these transporter inhibitors, such as MPP^+^, TEA, l-carnitine, pyrimethamine, and cimetidine (Tables 1, 2). In summary, these lines of evidence suggest that typical organic cation transporters which are expressed in the BCSFB are not involved in *p*-TA transport across the BCSFB. Other plasma membrane transporters for *p*-TA are known: i.e. several Na^+^-, Cl^−^-dependent Slc molecules, such as DAT and SERT. However, in the uptake study using isolated rat choroid plexus and TR-CSFB3 cells, [^3^H]*p*-TA uptake was not attenuated in the absence of extracellular Na^+^ and Cl^−^ (Tables 1, 2). The *K*_m_ value of human DAT- and SERT-mediated *p*-TA transport has been reported to be 1.7 and 52.7 μM, respectively [23, 24], which is different from the *K*_m_ value obtained for *p*-TA uptake by TR-CSFB3 cells. In addition, [^3^H]*p*-TA uptake by both isolated rat choroid plexus and TR-CSFB3 cells was not significantly changed in the presence of serotonin, a substrate of SERT (Tables 1, 2). Consequently, we conclude that typical organic cation transporters and the Na^+^- and Cl^−^-dependent Slc family do not play a role in apical *p*-TA transport at the BCSFB.

We showed that *p*-TA transport into TR-CSFB3 cells exhibited an oppositely-directed H^+^-gradient (Fig. 4). Cationic drug transport system(s) in several tissues and blood-central nervous system barriers have been proposed [37–43]. It has been reported that human intestinal epithelial cells transport several cationic drugs, such as bisoprolol and metoprolol, in an extracellular pH-dependent manner [37, 38]. Our previous reports have shown that nicotine is taken up into rat hepatocytes and lung via unidentified transport system(s) which recognize several cationic drugs and exhibit a H^+^/substrate antiport behavior [39, 40]. Moreover, it has been shown that H^+^/substrate antiport system(s) involve blood-to-brain transport across the BBB of cationic compounds and drugs, such as pyrilamine, oxycodone, and nicotine, although the molecular identification of the system(s) has not been reported [41–43]. *p*-TA uptake by isolated rat choroid plexus (Table 1) and TR-CSFB3 cells (Table 2) was significantly inhibited by cationic drugs which also inhibited the uptake of pyrilamine, oxycodone, and nicotine by an in vitro BBB model cell line [41, 42]. Thus, it is suggested that cationic drug-sensitive transport system(s), similar to the systems in the BBB, are present on the apical membrane of the BCSFB and are involved in *p*-TA elimination across the BCSFB from the CSF.

In patients with Parkinson’s disease and depression, monoamine oxidase inhibitors, such as selegiline, are regularly prescribed. It is widely known that patients who take *p*-TA-enriched foods exhibit neural side-effects such as migraine when receiving pharmacotherapy with monoamine oxidase inhibitors [44, 45]. As one reason for the above side-effects, the activation of noradrenergic signal transduction has been proposed by monoamine oxidase inhibition and, thus, an increase in the neural *p*-TA concentration. Many monoamine oxidase inhibitors, including selegiline, are cationic and lipophilic drugs. This study has demonstrated that *p*-TA elimination across the BCSFB from the CSF is inhibited by cationic and lipophilic compounds (Tables 1, 2). Thus, it is possible that the administration of cationic drugs causes an increase in *p*-TA in the brain via the inhibition of BCSFB-mediated *p*-TA elimination and exacerbates neuronal actions induced by excess *p*-TA. Further studies on the identification of *p*-TA transport molecule(s) which also recognize cationic drugs are needed, since this could help improve the pharmacotherapy of these patients in addition to increasing our understanding of the role of the BCSFB in homeostasis of *p*-TA levels in the brain. Moreover, it is considered that our findings about the in vitro characteristics of tyramine transport at the BCSFB (Tables 1, 2), such as inhibition by cationic drugs and promotion by the presence of several compounds (i.e., tyrosine, choline, MPP^+^, and TEA), are helpful for identifying the responsible molecule(s) for tyramine transport at the BCSFB.

## Conclusion

Our study demonstrates the greater contribution of *p*-TA elimination from the CSF to the clearance of *p*-TA from the brain parenchyma in vivo. From the transport studies using isolated choroid plexus and TR-CSFB3 cells, *p*-TA efflux transport at the BCSFB was shown to involve cationic drug-sensitive transport systems. These are distinct from typical plasma membrane organic cation transporters in the BCSFB. This study provides a new view of the role of the BCSFB in homeostasis of trace amines in the brain, including *p*-TA.

## Abbreviations

BBB: blood–brain barrier
BCSFB: blood-cerebrospinal fluid barrier
*C*_CSF_(*t*): concentration in CSF at time *t*
CSF: cerebrospinal fluid
DAT: dopamine transporter
DMSO: dimethyl sulfoxide
HEPES: 2-[4-(2-hydroxyethyl)-1-piperazinyl]ethansulfonic acid
*k*_e,CSF_: elimination rate constant from the CSF
*K*_d_: clearance of non-saturable transport
*K*_m_: Michaelis–Menten constant
MATE: multidrug and toxin extrusion
MPP^+^: 1-phenyl-4-phenylpyridinium
OCT: organic cation transporter
OCTN: organic cation/carnitine transporter
PMAT: plasma membrane monoamine transporter
*p*-TA: *para*-tyramine
[S]: the *p*-TA concentration in buffer
S.D.: standard deviation
S.E.M.: standard error of the mean
SERT: serotonin transporter
Slc: solute carrier
TR-CSFB: conditionally immortalized rat choroid plexus epithelial cells
*V*: uptake rate
*V*_d,CSF_: distribution volume in the CSF
